# A novel severe cerebral venous thrombosis rat model based on semi‐ligation combined with ferric chloride and thrombin

**DOI:** 10.1111/cns.13950

**Published:** 2022-08-23

**Authors:** Lipo Xiao, Xunming Ji, Haiping Zhao, Yumin Luo, Shuyuan Hu, Tingyu Zhao, Zeliang Hu, Jiangang Duan

**Affiliations:** ^1^ Department of Emergency, Xuanwu Hospital Capital Medical University Beijing China; ^2^ Department of Neurology and Intracranial Hypertension & Cerebral Venous Disease Center National Health Commission of the People's Republic of China, Xuanwu Hospital, Capital Medical University Beijing China; ^3^ Department of Neurology The People's Hospital of Qingxian Cangzhou China; ^4^ Cerebrovascular Diseases Research Institute and Department of Neurology Xuanwu Hospital, Capital Medical University Beijing China; ^5^ Department of Pathology, Xuanwu Hospital Capital Medical University Beijing China

**Keywords:** animal model, cerebral venous sinus thrombosis, semi‐ligation, severe, superior sagittal sinus

## Abstract

**Aims:**

An applicable cerebral venous sinus thrombosis (CVST) model is imperative for exploring its pathophysiology. We established a novel severe CVST model using semi‐ligation, ferric chloride, and thrombin.

**Methods:**

A total of 138 male Sprague–Dawley rats were randomly divided into semi‐ligation (*n* = 75) and non‐semi‐ligation (*n* = 63) groups. A sham group (*n* = 46) was also included. We compared short‐term and long‐term neurological and cognitive dysfunction, mortality rates, thrombus load, venous infarction volume, the blood–brain barrier permeability, brain water content, and microglia activation among the three groups.

**Results:**

Thrombi involving multiple venous sinuses appeared in all semi‐ligation rats within 2 days postoperatively. Compared with the non‐semi‐ligation group, short‐term and long‐term neurological dysfunction were more severe (*p* < 0.05), and thrombus weight, venous infarction volumes, and microglia activation were more significant (*p* < 0.05) in the semi‐ligation group. Further, the cognitive function of the semi‐ligation group significantly decreased (*p* < 0.05) on postoperative day 21. Cumulative mortality rates between the semi‐ligation and non‐semi‐ligation groups did not differ significantly.

**Conclusion:**

Semi‐ligation combined with ferric chloride and thrombin can produce a severe CVST model with multiple venous sinus involvement, which is suitable for short‐ and long‐term neurological and cognitive dysfunction assessment.

## Graphical abstract

Semi‐ligation combined with ferric chloride and thrombin can serve to produce a severe and stable CVST model with multiple venous sinuses involvement, and heavy thrombus load. Inflammation may be involved in the pathophysiology of severe CVST.

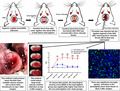

## 1 INTRODUCTION

Cerebral venous sinus thrombosis (CVST) is a special type of cerebrovascular disease that mainly affects young people,^1^, ^2^ especially pregnant and puerperium women,^3^ with an annual incidence of 13.0–15.7 per million.^4^ CVST accompanied by cerebral venous infarction or hemorrhage is categorized as a severe condition with a poor prognosis.^5^, ^6^ CVST can be treated through anticoagulation and surgical intervention; however, patients with severe CVST have a mortality rate of 34.2%.^7^ This high‐mortality rate may be attributed to the pathogenesis and pathophysiology of severe CVST being unclear. An applicable CVST model is not only paramount in the assessment of the pathogenesis and pathophysiology of severe CVST but is also suitable for preclinical experimental evaluation of novel therapeutic strategies for patients with severe CVST.^8^

Currently, methods for establishing a CVST model include complete ligation of the superior sagittal sinus (SSS) and thrombin injection,^9^, ^10^ balloon occlusion,^11^ photochemical induction,^12^ application of ferric chloride,^13^, ^14^ self‐made graft embolism,^15^, ^16^ and bipolar electrocoagulation.^17^, ^18^ However, these methods can rarely produce venous sinus thrombosis, cortical venous thrombosis, cerebral venous infarction, and hemorrhage simultaneously. Furthermore, thrombosis in most animal models rarely persists beyond 1 week.^9^, ^10^, ^11^, ^12^, ^13^, ^14^ Previous CVST models have induced thrombosis in only the SSS without disseminating to other venous sinuses. Particularly, current CVST models are incapable of producing thrombosis in multiple venous sinuses, which are present in 57% of patients with CVST.^4^ Ferric chloride can damage venous sinus endothelial cells through redox reactions, thereby inducing platelet activation and aggregation, followed by thrombus formation via a fibrin clot.^19^ However, ferric chloride alone, ferric chloride combined with thrombin, or ligation of the SSS combined with thrombin injection cannot induce multiple sinus thrombosis.^9^, ^10^, ^13^, ^14^ Therefore, it is imperative to establish a novel severe CVST model with multiple sinus thrombosis.

This study aimed to develop a novel severe CVST model with multiple sinus thrombosis through semi‐ligation combined with ferric chloride and thrombin. Semi‐ligation of the SSS can change blood flow status, ferric chloride can damage the SSS endothelium, and thrombin injection can cause blood hypercoagulability, effectively simulating the three elements of thrombosis.^19^ We evaluated this novel model in terms of neurological and cognitive dysfunction, body weight changes, cumulative mortality rate, thrombus load, venous cerebral infarction volume, blood–brain barrier (BBB) permeability, brain water content, histological changes, and immunofluorescence of microglia in the brain tissue.

## 2 METHODS

### 2.1 Animal preparation

The study protocol was approved by the Animal Experiments and Experimental Animal Welfare Committee of Capital Medical University (AEEI‐2020‐119) in Beijing, China, and was conducted in compliance with the institution's Animal Care and Use Committee regulations. The reporting of animal data in the current study followed the ARRIVE 2.0 guidelines.^20^ A total of 138 male Sprague–Dawley (SD) rats (280–350 g) were randomly divided into two groups: the semi‐ligation group (*n* = 75) and the non‐semi‐ligation group (*n* = 63). A sham group (*n* = 46) was also included in this study. Among 184 SD rats, 150 rats (*n* = 60 for the semi‐ligation group, *n* = 50 for the non‐semi‐ligation group, and *n* = 40 for the sham group) underwent short‐term postoperative assessment, including assessment of the neurological deficit, BBB permeability, brain water content, thrombus load, venous infarction volume, histological examination, and microglia activation. The remaining 34 rats (*n* = 15 for the semi‐ligation group, *n* = 13 for the non‐semi‐ligation group, and *n* = 6 for the sham group) were used for long‐term assessment, including assessment of neurological deficits, cumulative mortality rate, body weight, and cognitive function. The two protocols are shown in Supplementary Files 1 and 2, respectively. All the animals were maintained on an alternating 12‐h light/dark cycle with access to food and water ad libitum.

### 2.2 Application of the Pericam Perfusion Speckle Imager system

The Pericam Perfusion Speckle Imager (PSI) uses a 785 nm invisible laser for blood perfusion measurement of the cerebral ischemic model before, during, and after modeling. We selected a circular area in the middle of the SSS as the region of interest (ROI). We obtained the cerebral blood flow (CBF) in the ROI and investigated CBF changes during surgery to determine whether the semi‐ligation was successful and if the thrombus had been successfully induced. Additionally, it is helpful to monitor post‐surgical CBF changes in semi‐ligation and non‐semi‐ligation animals.

### 2.3 Thrombus induction

Rats were anesthetized using 5% enflurane and were maintained using 1–2% enflurane intraoperatively delivered in a mixture of 70% nitrous oxide and 30% oxygen through a face mask. The animals were placed in the prone position with their heads fixed at the midline using a stereotaxic frame (David Kopf Instruments, Tujunga, California, USA). Throughout the experiment, the temperature was maintained at 37 ± 0.5°C using a thermostatically controlled heating pad (Harvard Apparatus 50‐7061‐f, Holliston, MA, USA). A 1.5‐cm incision was made in the middle of each rat's head, and the subcutaneous tissue was separated to expose the skull. A longitudinal cranial window (10 × 4 mm), which exposed the SSS and bilateral cortex, was created between the bregma and lambda using a high‐speed dental drill (Strong‐207B, Saeshin, Busan, South Korea) through microscopic (Carl Zeiss, Inc., Berlin, Germany) observation. During the drilling process, the drill tip was continuously cooled with normal saline to avoid thermal injury in the dura mater and cortex. Subsequently, we turned on the laser speckle meter (PeriCam PSI System, Sweden) and aimed the camera at the SSS. CBF of the ROI in the SSS was detected and recorded.

In the semi‐ligation group, the SSS was rostrally and caudally semi‐ligated using an 8–0 polyamide suture (Ningbo Chenghe Micro Apparatus Factory, China). Subsequently, the laser speckle meter was used to detect CBF changes in the ROI after SSS semi‐ligation. The success of the semi‐ligation process was indicated by a CBF decrease to approximately 50% of that before semi‐ligation. A 7‐mm length of 3–0 silk thread was then dipped in 40% ferric chloride (Tianjin Zhiyuan Chemical Reagent Co., Ltd., China) and used to cover the SSS surface for 5 minutes in the absence of light. The field was then washed with normal saline after the silk thread was removed. Subsequently, thrombin (0.1 ml, 500 IU/mL; Chang Chun Lei Yunshang Pharmaceutical Co., Ltd., China) was injected into the sinus cavity of the ligated segment using a microinjector three times within 1 min. CBF after thrombin injection was significantly lower than CBF before ferric chloride and thrombin application, indicating successful thrombus induction.

In the non‐semi‐ligation group, the rats did not undergo semi‐ligation, and CBF of the ROI in the SSS was detected and recorded after SSS exposure. Other operations performed, including the external application of silk thread dipped in ferric chloride and thrombin injection, were identical to those performed in the semi‐ligation group. Subsequently, CBF of the ROI in the SSS was detected again and was found to be significantly lower than that after SSS exposure. In the sham group, the animals received only skull fenestration.

The surgical site was irrigated with normal saline, followed by sealing of the skull window with bone wax in each group. Postoperatively, each rat received a subcutaneous injection of ketoprofen (5 mg/kg) for analgesia.

### 2.4 Neurological and cognitive dysfunction evaluation

We used the neurological severity score (NSS)^21^ and rotarod test to estimate short‐term neurological function on days 1, 2, 3, 4, and 7 after modeling. In the rotarod test, all the rats were trained for three consecutive days and three times 1 day before surgery; the average time spent on the rod on the third day was considered the baseline. Rotarod speed accelerated from 4 to 40 rpm within 300 s. We tested each rat three times at different time points.

Among 184 SD rats, 34 rats underwent postoperative long‐term assessment for neurological deficits (days 1, 3, 5, 7, and 14), cognitive function (days 21 and 28), body weight (days 1, 3, 5, 7, 14, 21, and 28), and the cumulative mortality rate (days 1, 3, 5, 7, 14, 21, and 28). We adopted a discrimination index representing the evaluation tool of novel object recognition to assess cognitive dysfunction on days 21 and 28. The test process was reported previously.^22^

### 2.5 BBB permeability detection

Evans blue (EB, 2%, in phosphate‐buffered saline [PBS], 2 ml/kg; Sigma‐Aldrich) was administered intravenously in the tail vein on the postoperative day 2. Two hours after injecting successfully, the rats were sacrificed and PBS was perfused through their left ventricle. The brain tissue was separated and cut into 2‐mm sections to observe EB penetration. EB content was used to assess BBB permeability, as reported previously.^23^

### 2.6 Brain water content

On postoperative day 2, we evaluated the brain water content of the three groups. The rats were sacrificed, and the brains were removed and stripped of the dura mater, brain stem, and cerebellum. The cerebrum was placed in an oven at 110°C for 24 h. Brain water content was calculated using the formula ([wet brain weight − dry brain weight]/wet brain weight) and expressed as a percentage.

### 2.7 Comparisons of thrombus load and venous cerebral infarction volume

We evaluated thrombus load in the semi‐ligation, non‐semi‐ligation, and sham groups at different time points. Rats were sacrificed on postoperative days 1, 2, and 7. We opened the skull to observe the thrombus load with the naked eye after normal saline perfusion. The thrombus was then isolated for quantitative analysis. Next, the brain tissue was isolated for triphenyl tetrazolium chloride (TTC; Sigma‐Aldrich) staining. Brain slices (2 mm) were incubated with 2% TTC solution at 37°C for 20 minutes. We used image analysis software (Image J, 1.51j8, NIH, USA) to calculate the venous cerebral infarction volume for statistical analysis (infarct volume = area of infarct in square millimeters × thickness [2 mm]).

### 2.8 Immunofluorescence staining

We used Iba‐1 to label the microglia/macrophages in the tissue around the middle one‐third of the SSS, known as the parasagittal sinus brain tissue, of each group on postoperative days 1, 2, and 7. Brain tissue was extracted, dehydrated, and frozen. The frozen tissues were cut into 16 μm sections. The sections were immersed in mixed liquid (5% goat serum +0.5% Triton X‐100) for permeabilization and blocking after three washes with PBS, and then incubated with primary antibodies for microglia/macrophages (Iba‐1 1:500, GeneTex) overnight at 4°C. After three washes with PBS, sections were incubated with secondary antibodies (Alexa Fluor® 488‐conjugated goat anti‐rabbit, Abcam) for 1 h at 20°C ± 5°C. Finally, the sections were incubated with H‐1200 after three washes. Five consecutive slices from the middle of each specimen were cut, and five images were randomly captured from each slice under the microscope at 20× magnification. The mean gray value of microglia/macrophages in each image was calculated using ImageJ and used for statistical analysis (mean gray value = integrated density/area).

### 2.9 Histological examination of parenchymal tissue and the thrombus

Rats in the semi‐ligation group were sacrificed on postoperative days 2 and 7. Venous infarction tissue and thrombus were isolated and subjected to hematoxylin–eosin (HE) (Sigma‐Aldrich) staining.

### 2.10 Statistical analysis

All the data conformed to a normal distribution and are presented as mean ± standard deviation (SD). Comparisons between groups were performed using the paired samples *t*‐test or a one‐way analysis of variance (ANOVA), followed by the least significant difference test for pairwise comparisons. The values of the short‐term and long‐term NSS, rotarod test, cognitive function, and body weight among groups were analyzed using repeated‐measures ANOVA. Whether semi‐ligation was associated with mortality was assessed using the Kaplan–Meier survival analysis. All data analyses were performed using SPSS version 20.0 (SPSS, IBM, Armonk, NY, USA). A two‐tailed *p*‐value <0.05 was considered statistically significant.

## 3 RESULTS

### 3.1 Model success rate, mortality rate, hemorrhage rate, and seizure frequency in 1 week

The modeling success rate was 96.67% (58/60) and 84% (42/50) in the semi‐ligation group and non‐semi‐ligation group, respectively (*p >* 0.05). There was no significant difference in all‐cause mortality rates between the two groups; the mortality rates were 13.79% (8/58) and 4.76% (2/42) in the semi‐ligation and non‐semi‐ligation groups, respectively (*p >* 0.05). The cerebral hemorrhage rates of the semi‐ligation and non‐semi‐ligation groups in the first postoperative week were 84.44% (38/45) and 20.00% (7/35), respectively. However, none of the rats in the sham group (0/40) had a cerebral hemorrhage. Interestingly, three rats (5.17%, 3/58) in the semi‐ligation group, but none in the non‐semi‐ligation group, experienced general seizures (Video S1 legend is shown in Supplemental File 9).

### 3.2 Variation and comparison of cerebral blood flow diagram and curves during surgery

**FIGURE 1:**
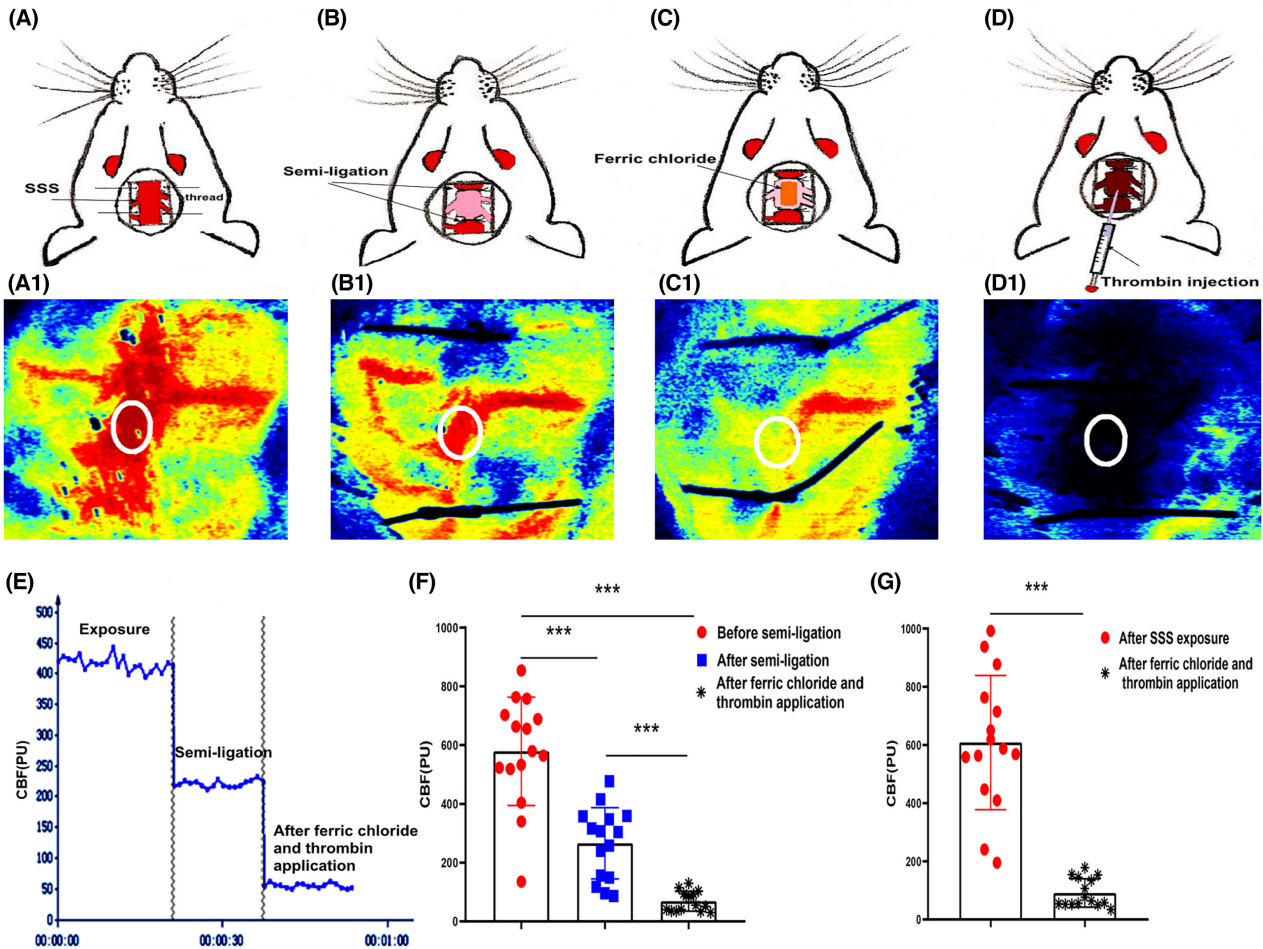
Ideograms and blood flow diagrams during surgery. (A–D) Ideograms respectively represent the superior sagittal sinus (SSS) exposure, SSS semi‐ligation, application of ferric chloride, and thrombin injection in sequence during modeling. The significantly red appearance of the SSS region of interest (ROI) (white circle) after the initial SSS exposure indicates sufficient blood flow (A). There is an obvious attenuation of the blood flow signal in the SSS ROI (white circle) after semi‐ligation, with the red signal decreasing by nearly half (B). Blood flow signal is further weakened in the ROI (white circle) after ferric chloride application (C). Red blood flow signal is replaced by blue after thrombin injection, indicating the complete interruption of blood flow and thrombosis in the SSS (D). (E) Representative graph of cerebral blood flow (CBF) changes in the ROI of the SSS during modeling in the semi‐ligation group. The horizontal and vertical axes represent the time and CBF values, showing the blood flow perfusion curve before semi‐ligation, after semi‐ligation, and after ferric chloride and thrombin application, respectively. This graph shows that CBF values before and after semi‐ligation, as well as after ferric chloride and thrombin application, are 400 PU, 200 PU, and 50 PU, respectively, proving the success of semi‐ligation and thrombotic induction. (F) CBF significantly decreases to approximately 50% after semi‐ligation compared with before semi‐ligation, and further decreases after ferric chloride and thrombin application in the semi‐ligation group (*n* = 15, ***p < 0.001). (G) CBF after the SSS exposure is also significantly reduced compared with that after the application of ferric chloride and thrombin in the non‐semi‐ligation group (*n* = 15, ***p < 0.001).

Figure 1A–D shows the process of preparing the model, including SSS exposure, SSS semi‐ligation, ferric chloride application, and thrombin injection. Figure 1A1–D1 shows CBF perfusion diagrams corresponding to different stages of surgery assisted by the laser speckle meter. Compared with the cerebral blood perfusion before semi‐ligation, red blood flow signal in the SSS ROI (white circle) after semi‐ligation was reduced by nearly half. After ferric chloride and thrombin application, the red blood flow signal in the ROI further decreased and disappeared, indicating complete blood flow interruption in the SSS after modeling. Figure 1E shows the corresponding cerebral blood perfusion curves during surgery in the semi‐ligation group. There were significant differences before and after semi‐ligation and after ferric chloride and thrombin application (578.76 ± 184.32 PU vs. 265.73 ± 121.24 PU vs. 68.29 ± 33.65 PU, *p* < 0.001, *n* = 15. PU = perfusion unit, vs = versus) (Figure 1F). In the non‐semi‐ligation group, there was also a significant difference between CBF after SSS exposure and after ferric chloride and thrombin application (520.28 ± 210.86 PU vs. 90.68 ± 46.50 PU, *p* < 0.001, *n* = 15) (Figure 1G). The quantitative analysis of CBF is shown in Supplementary File 3. However, the perfusion curve of the sham group remained relatively stable, and CBF did not change during surgery (*n* = 15)

### 3.3 Short‐term neurological deficit evaluation

**FIGURE 2:**
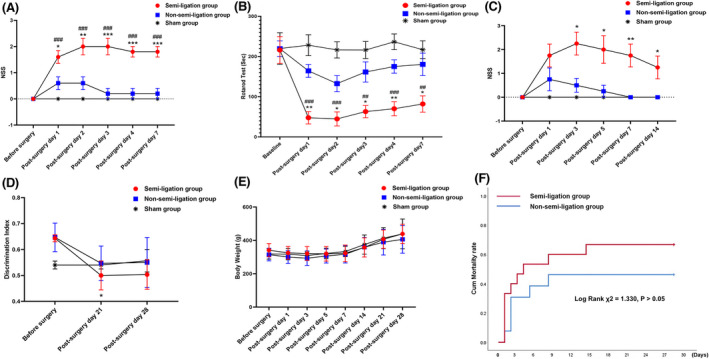
Comparisons of neurological, cognitive dysfunction, body weight, and survival assessments among the three groups. The short‐term neurological severity scores (NSS) differs significantly on days 1, 2, 3, 4, and 7 after modeling among the semi‐ligation, non‐semi‐ligation and sham groups (*: semi‐ligation vs. non‐semi‐ligation, *n* = 5 per group, *p < 0.05, **p < 0.01, ***p < 0.001; #: semi‐ligation vs. sham, *n* = 5 per group, ^###^ *p* < 0.001) (A). As for the short‐term rotarod test, rats in the semi‐ligation group stay on the rod shorter than did the non‐semi‐ligation and sham groups at the corresponding time points (*: semi‐ligation vs. non‐semi‐ligation, *n* = 5 per group, *p < 0.05, **p < 0.01; #: semi‐ligation vs. sham, *n* = 5 per group, ^##^ *p* < 0.01, ^###^ *p* < 0.001) (B). On postoperative day 14, the long‐term NSS of the semi‐ligation group remains higher than that of the non‐semi‐ligation group (*n* = 4 per group, *p < 0.05) (C). We adopted a discrimination index representing novel object recognition. Compared with the baseline, the discrimination index in the semi‐ligation group is significantly smaller (*n* = 3 per group *p* < 0.05) on postoperative day 21 and increased slightly on postoperative day 28 (*p >* 0.05). On the contrary, the baseline discrimination indexes do not differ significantly (*n* = 3 per group, *p >* 0.05) compared with those on postoperative days 21 and 28 in the non‐semi‐ligation and sham groups (D). The trends of body weight changes among the three groups are consistent at different time points after surgery, firstly decreasing and then increasing gradually within 28 days post‐surgery. The body weights do not differ significantly at the corresponding time points among the three groups (*n* = 4 per group, *p >* 0.05) (E). Survival analysis show that cumulative mortality rates between the semi‐ligation and the non‐semi‐ligation groups do not differ significantly (log rank χ^2^ = 1.330, *p >* 0.05, *n* = 15, *n* = 13) at each time point (F).

We selected 10 rats each from the semi‐ligation, non‐semi‐ligation, and sham groups for neurological function assessment. At various time points, the NSS in the semi‐ligation group was significantly higher than that of the other two groups (*p* < 0.001) (Figure 2A). Rats in the semi‐ligation group spent less time on the rod than those in the non‐semi‐ligation and sham groups at corresponding time points (*p* < 0.01) (Figure 2B **)**. Individual data of the NSS and rotarod test are shown in Tables S1 and S2 of Supplementary File 4, and original data for short‐term neurological deficits are provided in Tables S3 and S4 of Supplementary File 5.

### 3.4 Long‐term neurological and cognitive dysfunction, body weight, and survival assessments among the three groups

On the postoperative day 14, the NSS in the semi‐ligation group was still higher (*p* < 0.05) than that in the non‐semi‐ligation and sham groups (Figure 2C). Novel object recognition analyses indicated that cognitive function in the semi‐ligation group decreased significantly compared with baseline (0.500 ± 0.094 vs. 0.643 ± 0.023, *p* = 0.036) on postoperative day 21, and then gradually recovered (0.503 ± 0.098 vs. 0.643 ± 0.023, *p* = 0.109) until postoperative day 28. Cognitive function in the non‐semi‐ligation and sham groups did not differ significantly from baseline (0.547 ± 0.115 vs. 0.647 ± 0.095, *p* = 0.137; 0.540 ± 0.026 vs. 0.540 ± 0.026, *p* = 1.000) on day 21 and (0.550 ± 0.166 vs. 0.647 ± 0.095, *p* = 0.311; 0.557 ± 0.075 vs. 0.540 ± 0.026, *p* = 0.987) on day 28 (Figure 2D). The original data for long‐term NSS and the cognitive function assessment of the three groups are provided in Tables S5 and S6 of Supplementary File 5. The individual data points for long‐term NSS are provided in Table S11 of Supplementary File 6.

The body weights of the three groups showed a decreasing trend within 1‐week post‐surgery; this trend was greatest within 3 days and then started to increase gradually after 1 week. The body weights among the three groups did not differ significantly on days 1, 3, 5, 7, 14, 21, and 28 after surgery (*p >* 0.05, Figure 2E). The all‐cause mortality rates of the semi‐ligation group and non‐semi‐ligation group were 66.7% (10/15) and 46.2% (6/13), respectively, within 28 days (*p* = 0.249). Furthermore, the cumulative mortality rates of the semi‐ligation and non‐semi‐ligation groups did not differ significantly on postoperative days 1, 3, 5, 7, 14, 21, and 28 (log‐rank χ^2^ = 1.33, *p* = 0.249, Figure 2F). The Kaplan–Meier survival curve indicated that death mainly occurred within 5 days of surgery, especially 48 h after surgery in the semi‐ligation and non‐semi‐ligation groups. The original data for body weights and survival analysis of the three groups are also provided in Tables S7 and S8 of Supplementary File 5. The individual data points for body weights are provided in Table S12 of Supplementary File 6.

### 3.5 Comparisons of BBB permeability and brain water content

**FIGURE 3:**
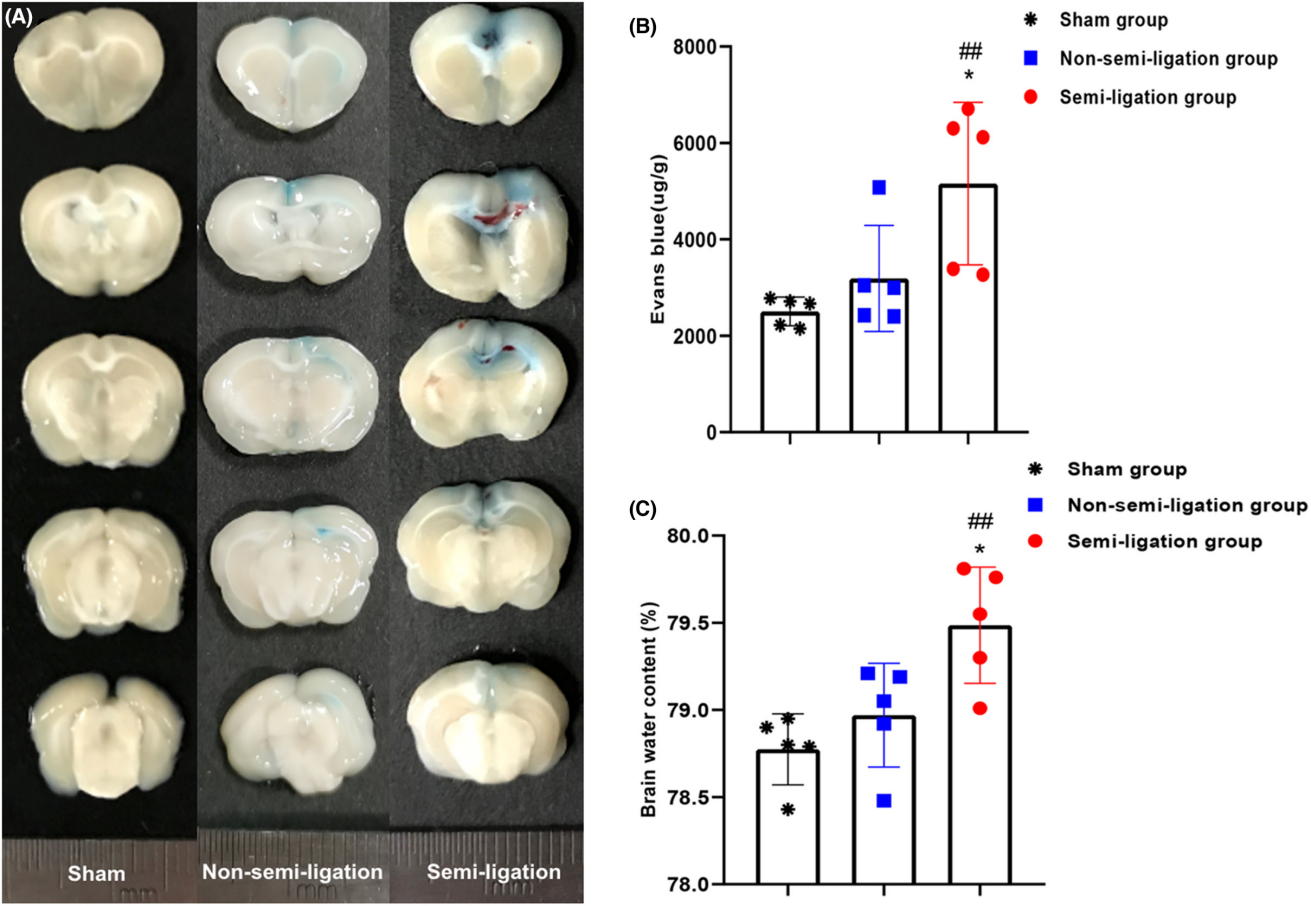
Comparisons of BBB permeability and brain water content among the three groups. Five rats from each group are used, respectively, for Evan Blue (EB) staining and brain water content evaluation on postoperative day 2. EB staining intensity of parasagittal sinus brain tissue in the semi‐ligation group is the most obvious among the three groups on postoperative day 2 (A). The blood–brain barrier permeability in the semi‐ligation group is significantly higher than that in the non‐semi‐ligation (*p < 0.05) and sham (^##^ *p* < 0.01) groups (B). Brain water content in the semi‐ligation group is also significantly higher than that in the other two groups (*p < 0.05; ^##^ *p* < 0.01) (C).

On the postoperative day 2, we selected five rats in each group for EB staining and a further five for brain water content assessment. EB exudation in the brain tissue of the semi‐ligation group was significantly heavier than that of the non‐semi‐ligation and sham groups (Figure 3A). EB staining intensity in the brain tissue of the semi‐ligation group was significantly higher than that of the other two groups (5157.37 ± 1683.97 mg/g vs. 3190.18 ± 1099.78 mg/g vs. 2505.60 ± 297.30 mg/g, *p* < 0.05) (Figure 3B **)**. Similarly, the brain water content of the semi‐ligation group was significantly higher than that of the non‐semi‐ligation and sham groups (79.49 ± 0.33% vs. 78.97 ± 0.30% vs. 78.77 ± 0.20%, *p* < 0.01) (Figure 3C **).**

### 3.6 Comparisons of thrombus load, venous infarction volume, and thrombus weight

**FIGURE 4:**
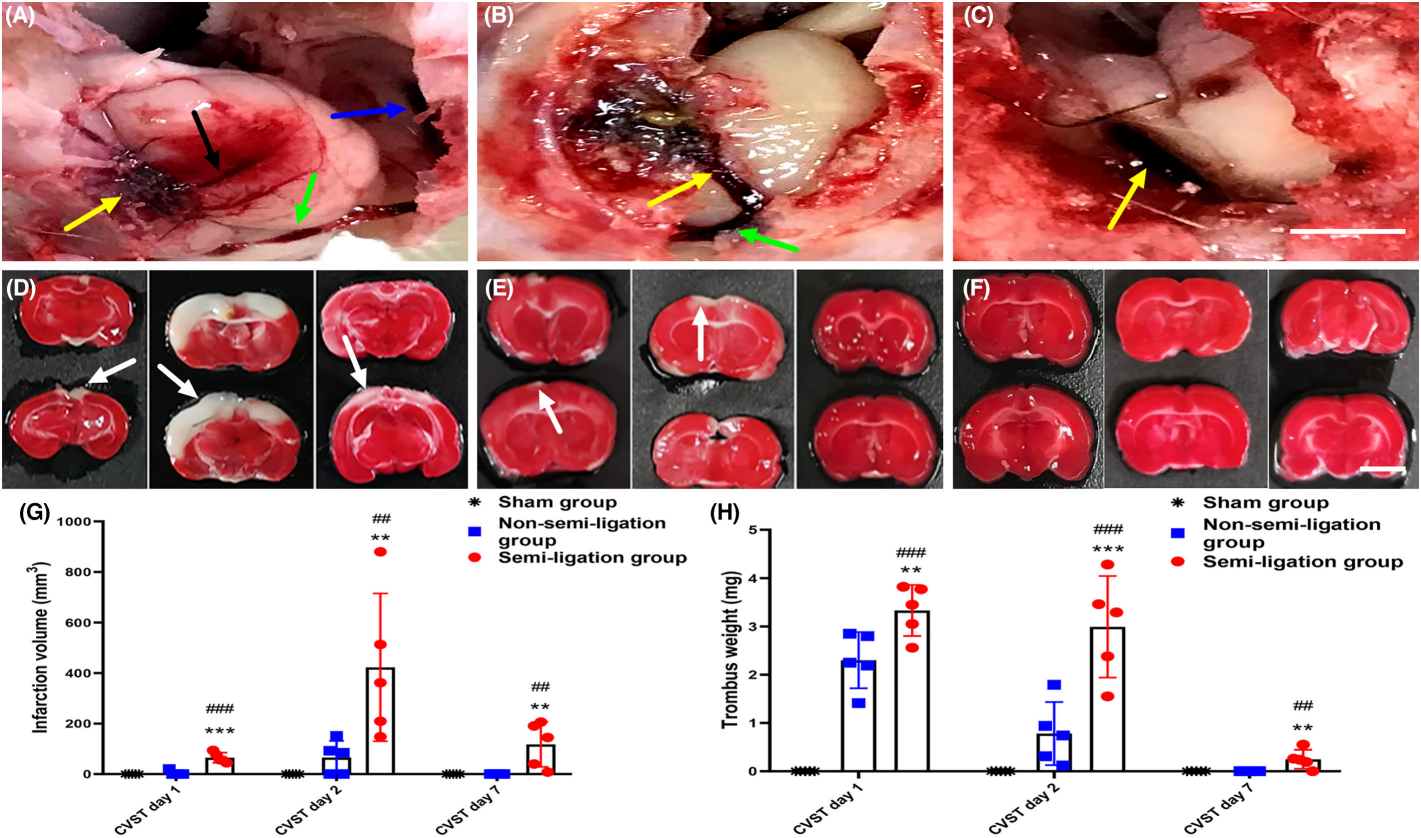
Thrombus load in the semi‐ligation group, and comparisons of venous cerebral infarction volumes and thrombus weights among the three groups. In the semi‐ligation group, thrombus load is demonstrated in A–C at different time points after surgery. (A) Thrombosis disseminates into the superior sagittal sinus (SSS) (yellow arrows), cortical vein (white arrows), right transverse sinus (green arrows), and right internal jugular vein (blue arrows) on postoperative day 1. (B) Thrombi in the SSS (yellow arrow) and left transverse sinus (green arrow) on postoperative day 2. The thrombus remains in the SSS (yellow arrow) on postoperative day 7 (C). The range of venous infarction (white arrow) in the semi‐ligation group is larger than that in the non‐semi‐ligation and sham groups at different postoperative time points (D–F). Venous infarction volumes in the semi‐ligation group are also significantly larger than that in the other two groups at the corresponding time points (*p* < 0.05). The venous infarction volumes in the semi‐ligation group are also significantly larger than that in the non‐semi‐ligation and sham groups on postoperative days 1, 2, and 7 (*: semi‐ligation group vs. non‐semi‐ligation group, #: semi‐ligation group vs. sham group, ^**^ *p* < 0.01, ^***^ *p* < 0.001, ^##^ *p* < 0.01, ^###^ *p* < 0.001, G). Thrombus weights in the semi‐ligation group are significantly heavier than that in the non‐semi‐ligation and sham groups on postoperative days 1, 2, and 7 (*: semi‐ligation vs. non‐semi‐ligation, #: semi‐ligation vs. sham, **p < 0.01, ***p < 0.001, ^##^ *p* < 0.01, ^###^ *p* < 0.001, H).

In the semi‐ligation group, a heavy thrombus load developed in multiple venous sinuses of all rats (5/5) on the postoperative day 1. The induced thrombi were disseminated to the transverse sinus, internal jugular vein, and even extended to the cortical veins (Figure 4A). On postoperative day 2, thrombosis involving multiple sinuses was present in all animals (5/5) (Figure 4B). The thrombus remained in the SSS in four rats (4/5) on postoperative day 7 (Figure 4C). In the non‐semi‐ligation group, thrombosis involving the SSS and transverse sinus presented successively in two (2/5) and one (1/5) rats on postoperative days 1 and 2, but the thrombi in all rats (5/5) were completely dissolved on postoperative day 7 (not shown). Thrombosis was not observed in the sham group at any time point (not shown).

After evaluating the thrombus load, we observed the venous infarction volume at different time points in the three groups using TTC staining. The venous cerebral infarction volume in the semi‐ligation group started to increase on postoperative day 1, reached a peak on day 2, and decreased on day 7 (Figure 4D). Similarly, the venous cerebral infarction volume in the non‐semi‐ligation group began increasing on postoperative day 1 and reached a peak on day 2, but no cerebral infarction was observed on day 7 (Figure 4E). No venous infarction was observed at all three time points in the sham group (Figure 4F). At the corresponding time points, the venous infarction volumes in the semi‐ligation group were also significantly larger than that in the other two groups (65.08 ± 19.97 mm^3^ vs. 4.04 ± 9.03 mm^3^ vs. 0.0 ± 0.0 mm^3^, *p* < 0.001; 422.33 ± 292.57 mm^3^ vs. 65.68 ± 65.33 mm^3^ vs. 0.0 ± 0.0 mm^3^, *p* < 0.01; 117.79 ± 89.48 mm^3^ vs. 0.00 ± 0.00 mm^3^ vs. 0.0 ± 0.0 mm^3^, *p* < 0.01) (Figure 4G). The comparison of thrombus weight was consistent with the comparison of venous infarction volume. Thrombus weights in the semi‐ligation group were also significantly heavier than that in the other two groups on postoperative days 1, 2, and 7 (3.33 ± 0.53 mg vs. 2.30 ± 0.58 mg vs. 0.0 ± 0.0 mg, *p* < 0.001; 2.99 ± 1.05 mg vs. 0.78 ± 0.65 mg vs. 0.0 ± 0.0 mg, *p* < 0.001; 0.25 ± 0.20 mg vs. 0.00 ± 0.00 mg vs. 0.0 ± 0.0 mg, *p* < 0.01) (Figure 4H). The data analyses for thrombus weights and infarction volumes among the three groups are provided in Tables S13 and S14 of Supplementary Files 7, respectively. The original data for thrombus load and infarction volumes are also shown in Supplementary File 5.

### 3.7 Comparisons of the inflammatory response trend among the three groups by microglia/macrophage staining

**FIGURE 5:**
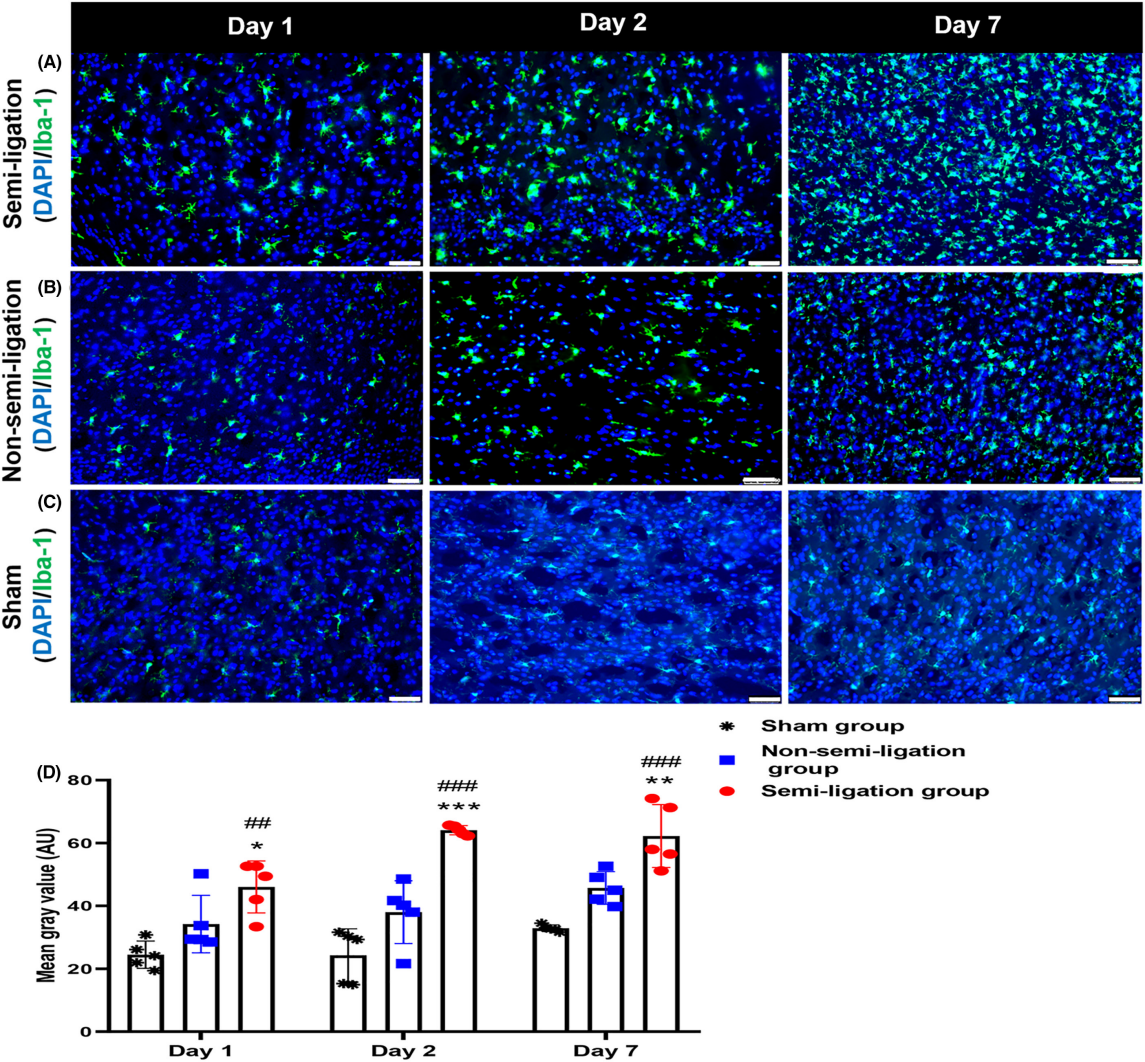
Comparisons of microglia/macrophage activation at different time points among the groups. (A–C) Immunofluorescence staining of microglia/macrophages (green, Iba‐1) and cell nuclei (blue, DAPI) in the parasagittal sinus brain tissue of the semi‐ligation, the non‐semi‐ligation, and sham groups on postoperative days 1, 2, and 7 (scale bar: 50 μm), respectively. (D) The mean gray values of microglia/macrophages in the semi‐ligation group are larger than those in the non‐semi‐ligation and sham groups on postoperative day 1 (*: semi‐ligation vs. non‐semi‐ligation, #: semi‐ligation vs. sham, *p < 0.05, ^##^ *p* < 0.01), day 2 (^***^ *p* < 0.001, ^###^ *p* < 0.001), and day 7 (^**^ *p* < 0.01, ^###^ *p* < 0.001). In the non‐semi‐ligation group, the mean gray values of microglia/macrophages are higher than those in the sham group on postoperative day 2 (*p* < 0.05) and day 7 (*p* < 0.01).

We selected five rats from each group for microglia immunofluorescence analysis on postoperative days 1, 2, and 7. Microglia activation (green) appeared on day 1, reached a peak on day 2, and lasted until 7 days in the parasagittal sinus brain tissue of the semi‐ligation group (Figure 5A). Similarly, microglia/macrophage activation also appeared on day 1, increased on day 2, and reached a peak on day 7 in the non‐semi‐ligation group **(**Figure 5B). However, microglia/macrophage activation in the brain tissue of the sham group remained stable on postoperative days 1 and 2, and slightly increased on day 7 (Figure 5C). The mean gray values of microglia/macrophages in the parasagittal sinus brain tissue of the semi‐ligation group were significantly greater than that of the other two groups on days 1, 2, and 7 (46.02 ± 8.27 AU vs. 34.22 ± 9.16 AU vs. 24.46 ± 4.32 AU, *p* < 0.01; 64.09 ± 1.47 AU vs. 38.01 ± 9.98 AU vs. 24.28 ± 8.42 AU, *p* < 0.001; 62.21 ± 9.98 AU vs. 45.71 ± 5.19 AU vs. 32.88 ± 1.03 AU, *p* < 0.001, AU = arbitrary units) (Figure 5D).

### 3.8 Histological changes of the thrombus and venous infarction in the semi‐ligation group

**FIGURE 6:**
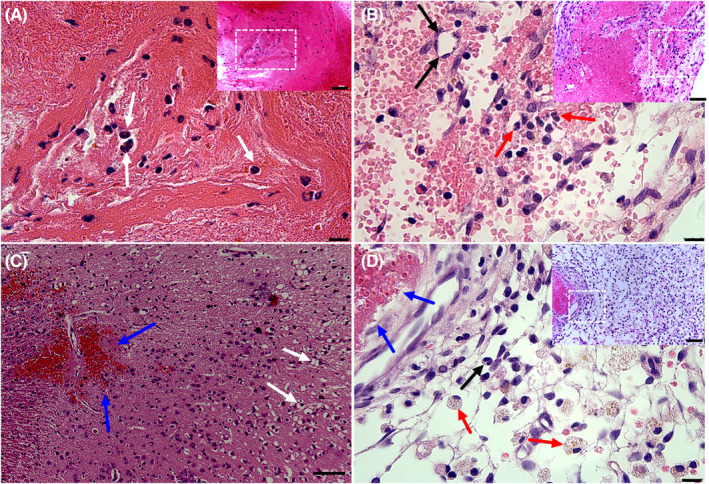
Histological changes of thrombus and venous infarction in the semi‐ligation group. (A and B) Histological changes in the superior sagittal sinus thrombus. Hematoxylin–eosin (HE) staining shows a thrombus filled with red blood cells, fibrin, and neutrophils (white arrow) on postoperative day 2 (A). Microvascular (black arrow) is observed in the thrombus by postoperative day 7; moreover, there is apparent neutrophil infiltration (red arrow) (B). (C and D) Histological changes in venous infarction tissue. Extended tissue spaces (white arrow), disorganized cell arrangement, and scattered microbleeds are visible (blue arrow) in the venous infarction area on postoperative day 2 (C). Enlarged intercellular spaces, increased phagocytes (red arrow), tissue loosening, venous thrombus (blue arrow), and some neutrophil infiltration (black arrow) are observed in venous infarction tissue on postoperative day 7 (D). Scale bars = 10 μm (A, B, and D), 100 μm (C), 50 μm (micrograph in A, B, and D).

We selected five rats in the semi‐ligation group for HE staining on postoperative days 2 and 7. On day 2, numerous red blood cells, fibrin, and neutrophils were observed in the thrombus (Figure 6A). One week post‐surgery, microvascular and numerous neutrophils were observed in the thrombus (Figure 6B). Moreover, tissue edema and scattered microbleed foci were noted in the venous infarction area on postoperative day 2 (Figure 6C). After 1 week, there were enlarged intercellular spaces, phagocytes, and neutrophil infiltration in the infarction area (Figure 6D).

## 4 DISCUSSION

We established a novel severe CVST model with multiple sinus involvement capable of mimicking the pathophysiology of severe human CVST. Compared with the non‐semi‐ligation group, short‐term and long‐term neurological dysfunction, thrombus load, cerebral infarction volume, BBB permeability damage, brain water content, and microglia/macrophage activation in the semi‐ligation group were more severe, heavier, and more significant, respectively. However, mortality rates and body weight between semi‐ligation and non‐semi‐ligation groups did not differ significantly within 28 days.

Compared with previous models, this novel model has several advantages. First, the semi‐ligation method can decrease SSS blood flow without completely occluding it, meaning that the assessment of treatment strategies is not affected. Li et al.^10^ established a CVST model using a combination of permanent ligation, direct thrombin injection, and temporary carotid artery occlusion. The advantages of Li's model included thrombus formation in the SSS and cortical venous thrombosis; however, permanent ligation impeded the assessment of therapeutic strategies. Second, to avoid cortical tissue injury, we used a 7‐mm length 3–0 silk thread rather than filter paper strips. Rottger et al.^13^ established a CVST model via topical application of filter paper strips soaked with ferric chloride. Despite being a simple method, the induced thrombus was short‐lasting and completely dissolved and recanalized within 1 week. Therefore, this model is unsuitable for long‐term observation. For increased stability of the induced thrombus, Wei et al.^14^ induced SSS thrombosis by topical application of ferric chloride and thrombin injection. Although this model improved stability, the application of filter paper strips soaked with ferric chloride inevitably caused a cortical injury. Recently, Bourrienne et al.^24^ induced a CVST model by injecting self‐made in vitro autologous thrombus into mouse SSS and ligating the bilateral external jugular veins. Although it can also induce pathological changes of ischemia and hemorrhage, and the thrombus can last for 1 week without obvious recanalization, the thrombus formed in vitro is still different from that formed in the body, and it cannot simulate the entire pathophysiology of CVST. The ideal animal model of CVST should have three critical features. First, it should represent the pathophysiological process of human CVST. Second, pathological changes, including venous sinus thrombosis, cortical venous thrombosis, and intracranial hemorrhage, should be observed simultaneously. Third, the model should provide a research platform for developing and testing novel therapies.^25^ In summary, the pathophysiological process of thrombosis in our model is closer to that of human CVST.

TTC findings revealed that the volume of cerebral venous infarction in the semi‐ligation group increased on postoperative day 1, reached a peak on day 2, and decreased on day 7; neurological dysfunction was also the most severe on day 2, and then gradually recovered. These results are consistent with previous observations.^12^ Moreover, our results suggest that thrombus load was heavy on postoperative days 1 and 2, and the least on day 7. Thus, we speculated that thrombus recanalization could save penumbrae by improving regional hemodynamics. Aguiar de Souse et al.^26^ reported that venous recanalization starts in the first 8 days of anticoagulation therapy in most CVST patients and is associated with the reversal of early cerebral venous infarction.

The mortality rate of severe cerebral edema was 13.79% in our model, which is similar to that of severe CVST in humans.^7^ Seizures are a clinical feature for approximately 12–31.9% of CVST patients and is seen especially in patients with severe CVST with cortical venous thrombosis and venous cerebral infarction.^27^, ^28^ Seizures were present in our novel model, with an incidence of 5.17%, which had not been reported in previous animal models of CVST.^9^, ^10^, ^11^, ^12^, ^13^, ^14^, ^15^, ^16^, ^17^, ^18^, ^23^ Therefore, our novel model is more suitable for simulating severe CVST. Our semi‐ligation group model does not increase the long‐term mortality rate compared with the non‐semi‐ligation group model and has cognitive dysfunction on postoperative day 21; thus, it can be used for short‐term and long‐term observation and research.

Microglia are resident macrophages of the central nervous system (CNS).^29^ It is currently believed that microglia originating from primitive progenitor cells in the yolk sac are the resident immune cells of the CNS and the first line of CNS defense.^29^, ^30^ Microglia activation is the first step of the inflammatory response in the brain, followed by infiltration and activation of immune cells, such as neutrophils, macrophages, T cells, and natural killer cells, among others.^31^ Hu et al.^32^ studied microglia activation in an animal model of arterial stroke, and Rashad et al.^33^ proved microglia activation after CVST with a non‐severe CVST model. Our study observed microglia activation at 1, 2, and 7 days after CVST. The activation of microglia in the semi‐ligation group peaked on postoperative day 2, when the infarction volumes were largest, the mortality rate was highest, and neurological dysfunction was most severe. Therefore, we speculated that inflammation activation may be positively correlated with infarct volume, mortality rate, and neurological dysfunction, but further research is needed. These results indicate that inflammation might participate in the pathophysiological mechanism of severe CVST, which is consistent with the findings of Nagai et al.^34^ Coincidentally, our recent research showed that the inflammatory response after CVST was related to admission severity and poor prognosis at discharge.^35^ Our latest study revealed that venous infarction was associated with inflammation.^36^ However, to determine the duration of the inflammatory response, whether the activated microglia are M1 type, M2 type, or both, and their changes over time, further research is needed. Whether the activation of microglia in the brain tissue and the infiltration of neutrophils in the thrombus are related or have a synergistic effect is also unclear, and further research is warranted.

Anticoagulation is still recommended as the gold standard for CVST according to treatment guidelines issued by the European Federation of Neurological Societies in 2017.^37^ Ding et al.^38^ found batroxobin can promote venous sinus recanalization and attenuate CVST‐induced stenosis. Therefore, our novel CVST model may be used to further explore the effectiveness, safety, and mechanism of batroxobin in severe CVST therapy. Moreover, our study suggested that inflammation might be involved in the pathophysiology of severe CVST. Hence, this novel CVST model may be used to further study the relationship between inflammation and severe CVST and the efficacy evaluation of anti‐inflammatory treatment strategies. Wu et al.^39^ designed an artery and venous sinus occlusion image score (AVOIS) to evaluate occlusive cerebral arteries and venous diseases. AVOIS provided a novel method for evaluating thrombosis in cerebral venous sinus and is highly effective and accurate in predicting poor outcomes. Therefore, we can use AVOIS to evaluate the level of venous sinus obstruction and the condition of prognosis for severe CVST in our future experiments.

This study has several limitations. First, most of the published studies on the CVST animal model, including the present study, used males rather than females. However, many recent studies reported that sex differences exist in brain vessels,^40^ cerebral blood flow,^41^, ^42^ venous disruption, venous thrombosis,^43^, ^44^ responses to stroke,^45^, ^46^ and stroke outcomes.^47^ CVST occurs more frequently in females, but the incidence rate in males is increasing annually.^48^ For the comparability of experimental data, we selected only male rats in this study; therefore, we will use both male and female rats to conduct a severe CVST model in the future. Second, we observed brain water content and the blood–brain barrier permeability at only one time point; therefore, their changes over time are unknown. However, this study also has several appreciable strengths. First, to the best of our knowledge, the novel severe CVST model is closer to the pathophysiological process of human CVST than existing models. Second, this is the first severe CVST model study to simultaneously assess short‐term and long‐term neurological and cognitive dysfunction. Finally, the modeling method used does not increase the mortality of severe CVST; thus, it can benefit research to understand the mechanism and treatment strategies of severe CVST.

In conclusion, semi‐ligation combined with ferric chloride and thrombin can serve to produce a severe and stable CVST model with the involvement of multiple venous sinuses, heavy thrombus load, severe neurological dysfunction, and cognitive dysfunction. This novel model can be used for short‐term or long‐term preclinical studies on severe CVST. Moreover, as inflammation may be involved in the pathophysiology of severe CVST, this model is expected to become an animal model more closely resembling the pathogenesis of severe CVST in humans.

## AUTHOR CONTRIBUTIONS

All the authors had full access to all study data and take responsibility for the integrity of the data and the accuracy of the data analyses. Study conception, design, and data analysis: Jiangang Duan. Material preparation, model establishment, data collection, and analysis: Lipo Xiao, Shuyuan Hu. Discussion: Xunming Ji, Haiping Zhao, Yumin Luo, Tingyu Zhao, Zeliang Hu, and Jiangang Duan. The first draft of the manuscript was written by Lipo Xiao and all the authors commented on previous versions of the manuscript.

## FUNDING INFORMATION

This study was supported by the Beijing Natural Science Foundation (No.7182064).

## CONFLICT OF INTEREST

None.

## Supporting information


**Video S1** Supporting Information

**Appendix S1** Supporting Information

## Data Availability

The data that support the findings of this study are available from the corresponding author upon reasonable request.
