# Supplementary material for: A novel severe cerebral venous thrombosis rat model based on semi‐ligation combined with ferric chloride and thrombin
Source: CNS Neurosci Ther. 2022 Aug 23;28(12):2129–40. doi: 10.1111/cns.13950 (PMC9627376; doi:10.1111/cns.13950)
Supplement: Supplementary file 2 — Appendix S1 Supporting Information [file CNS-28-2129-s002.docx]

**Supplementary 1**

**Short-term experimental design protocol**

Male Sprague-Dawley rats (n=150)

10 rats died

10 rats died

Non-semi-ligation group (n=50)

Semi-ligation group (n=60)

Sham group (n=40)

Post modeling

Thrombus Load and Venous Infarction Volume (n=5, per group) within 1 week

NSS and Rotarod test (n=5, per group) within 1 week

Microglia activation (n=5, per group) within 1 week

HE staining (n=5, only for Semi-ligation group) on days 2 and 7

BBB permeability (n=5, per group) on day 2

Brain water content (n=5, per group) on day 2

Among 10 dead rats in the semi-ligation group, 2 rats died during the operation and 8 rats died within 1 week after surgery. Among another 10 dead rats in the non-semi-ligation group, 8 rats died during the operation and 2 rats died within 1 week after surgery.

**Supplementary 2**

**Long-term experimental design protocol**

Male Sprague-Dawley rats (n=34)

Sham (n=6)

Non-semi-ligation (n=13)

Semi-ligation (n=15)

Cumulative mortality rates within 28 days after modeling

Neurological dysfunction within 14 days after modeling

Novel object recognition assessment before surgery and on day 21 and 28 after modeling

Body weight assessment within 28 days after modeling

34 male SD (Sprague-Dawley, SD) rats were used for post-operation long-term assessment, including the neurological deficit (days 1, 3, 5, 7, and 14), cognitive function (days 21 and 28), body weight (days 1, 3, 5, 7, 14, 21, and 28), and cumulative mortality rate (days 1, 3, 5, 7, 14, 21, and 28).

**Supplementary 3**

**Graph S1**


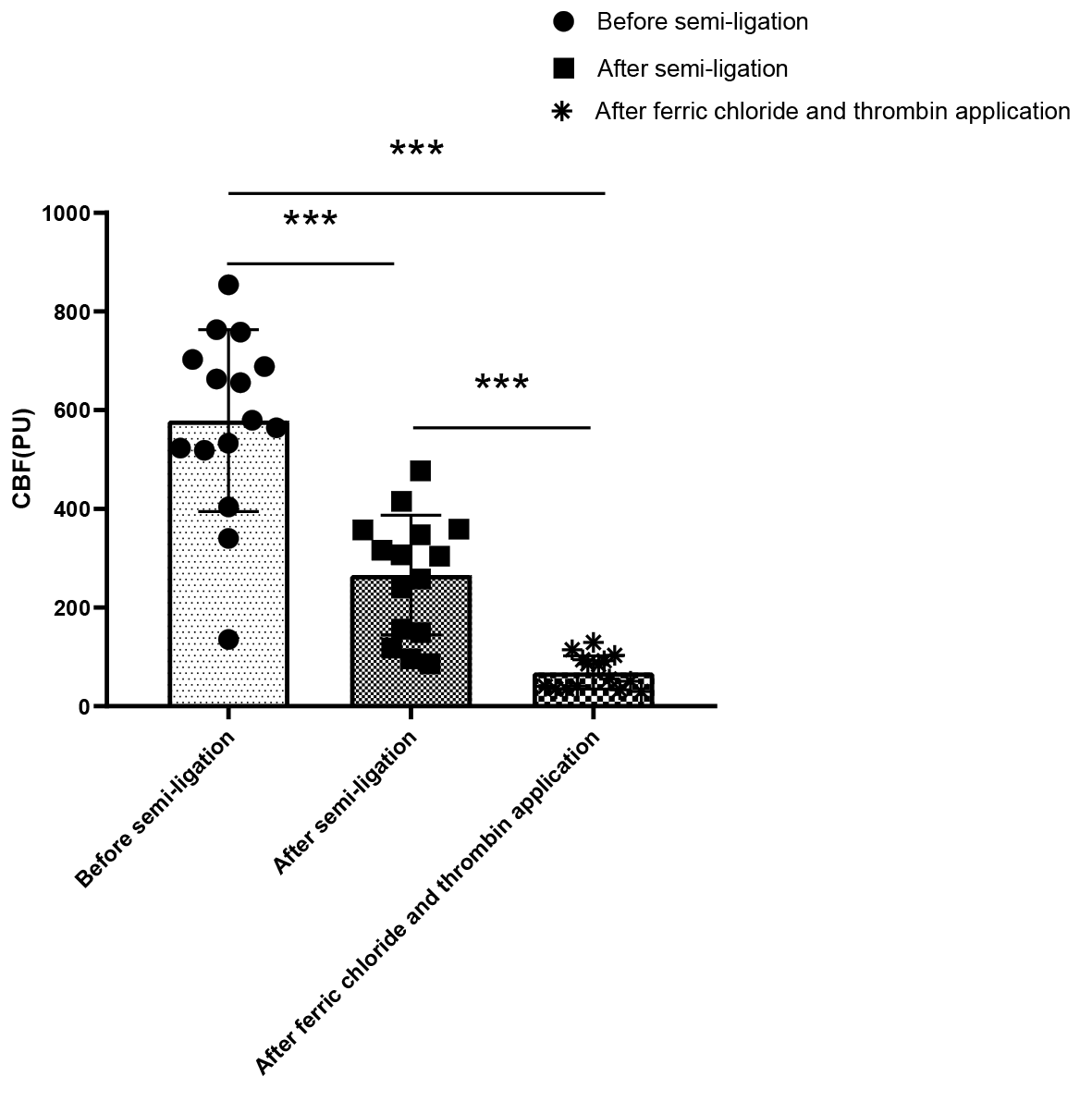


As shown in the supplementary graph S1, in the semi-ligation group, the cerebral blood flow (CBF) significantly decreased to 50% after semi-ligation (265.73 ± 121.24 PU), compared with before semi-ligation (578.76 ± 184.32 PU), and further decreased after ferric chloride and thrombin application. There were significant differences before and after semi-ligation, and after ferric chloride and thrombin application (n=15, *** *P*＜0.001).

**Graph S2**


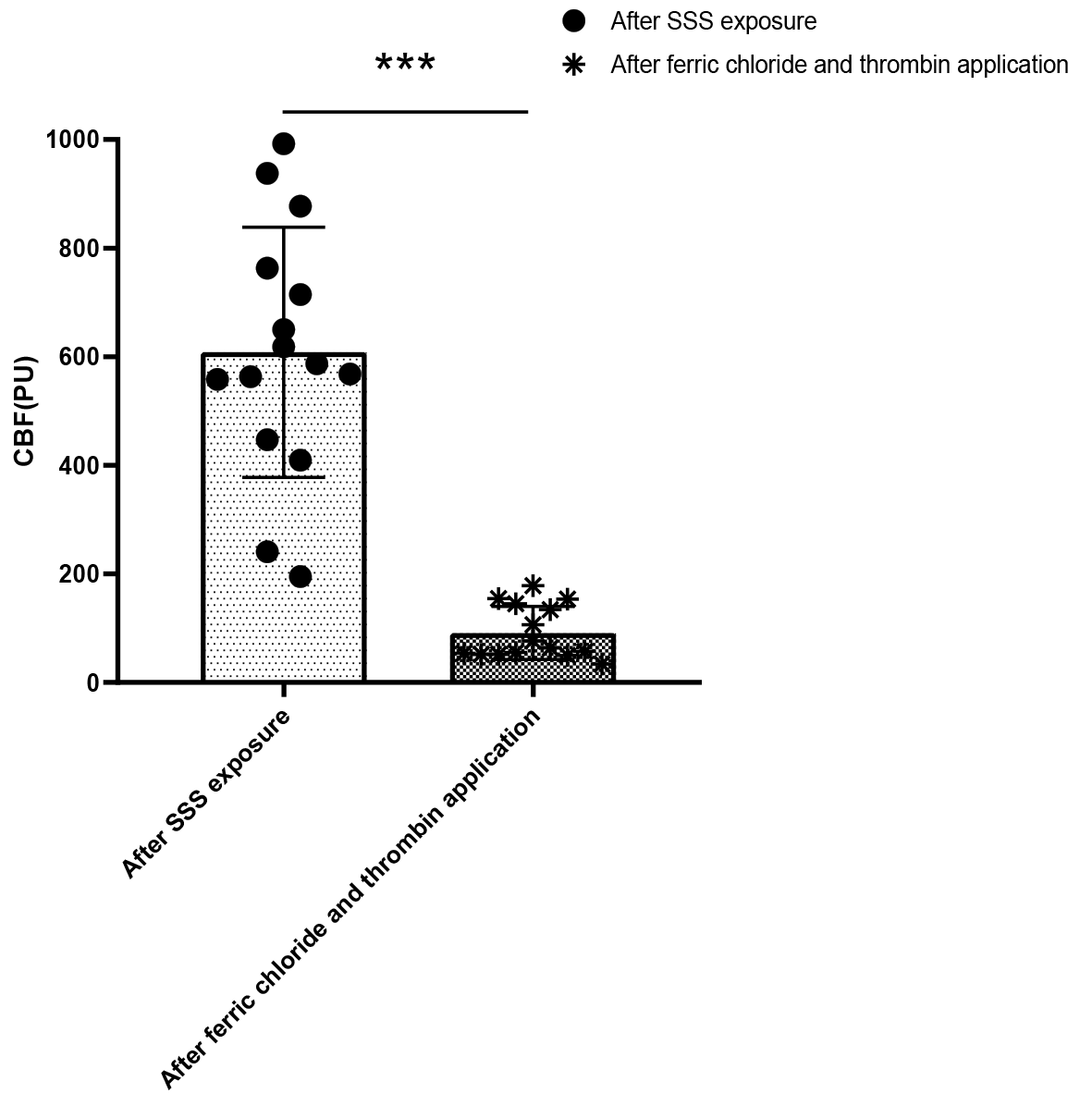


As shown in the supplementary graph S2, in the non-semi-ligation group, the cerebral blood flow (CBF) after ferric chloride and thrombin application (90.68 ± 46.50 PU) was obviously reduced compared with the CBF after superior sagittal sinus (SSS) exposure (520.28 ± 210.86 PU) (n=15, *** *P*＜0.001).

**Supplementary 4**

Table S1. Short-term Neurological severity scores (NSS) at different time points in the semi-ligation, non-semi-ligation and sham groups.

|  | Semi-ligation  (n=5) | Non-semi-ligation  (n=5) | Sham  (n=5) | F | P |
| --- | --- | --- | --- | --- | --- |
| Day 1 | 1.60±0.55 | 0.60±0.55 | 0±0 | 16.333 | 0.000 |
| Day 2 | 1.80±0.71 | 0.60±0.55 | 0±0 | 19.750 | 0.000 |
| Day 3 | 2.00±0.71 | 0.20±0.45 | 0±0 | 26.000 | 0.000 |
| Day 4 | 1.80±0.45 | 0.20±0.45 | 0±0 | 36.500 | 0.000 |
| Day 7 | 1.80±0.45 | 0.20±0.45 | 0±0 | 36.500 | 0.000 |

Values are expressed as mean ± standard deviation (SD)

At various time points, the NSS in the semi-ligation group were significantly higher than that of the non-semi-ligation and sham groups.

|  | Semi-ligation  (n=5) | Non-semi-ligation  (n=5) | Sham  (n=5) | F | P |
| --- | --- | --- | --- | --- | --- |
| Baseline | 216.11±74.82 | 219.45±43.34 | 219.93±87.99 | 0.004 | 0.996 |
| Day 1 | 47.71±34.16 | 164.30±36.02 | 228.21±58.01 | 21.55 | 0.000 |
| Day 2 | 44.87±39.01 | 132.59±44.72 | 216.53±45.07 | 19.898 | 0.000 |
| Day 3 | 63.03±34.25 | 161.49±56.11 | 216.25±47.50 | 13.75 | 0.001 |
| Day 4 | 70.20±39.00 | 175.11±37.33 | 236.64±43.67 | 22.03 | 0.000 |
| Day 7 | 81.94±45.40 | 180.51±62.30 | 216.99±49.21 | 8.75 | 0.005 |

Table S2. Short-term Rotarod test results (Sec) at different time points in the semi-ligation, non-semi-ligation and sham groups.

Values are expressed as mean ± standard deviation (SD)

The periods that rats spent on the rod in the semi-ligation group were shorter than the non-semi-ligation and sham groups at corresponding time points

|  | **Before surgery** | **Post-surgery day 1** | **Post-surgery day 2** | **Post-surgery day 3** | **Post-surgery day 4** | **Post-surgery day 7** |
| --- | --- | --- | --- | --- | --- | --- |
| Semi-ligation | 0 | 1 | 2 | 2 | 2 | 2 |
| Semi-ligation | 0 | 2 | 2 | 2 | 2 | 2 |
| Semi-ligation | 0 | 2 | 3 | 3 | 2 | 2 |
| Semi-ligation | 0 | 1 | 1 | 1 | 1 | 1 |
| Semi-ligation | 0 | 2 | 2 | 2 | 2 | 2 |
| Non-semi-ligation | 0 | 1 | 1 | 0 | 0 | 0 |
| Non-semi-ligation | 0 | 0 | 0 | 0 | 0 | 1 |
| Non-semi-ligation | 0 | 1 | 1 | 0 | 0 | 0 |
| Non-semi-ligation | 0 | 1 | 1 | 1 | 1 | 0 |
| Non-semi-ligation | 0 | 0 | 0 | 0 | 0 | 0 |
| Sham | 0 | 0 | 0 | 0 | 0 | 0 |
| Sham | 0 | 0 | 0 | 0 | 0 | 0 |
| Sham | 0 | 0 | 0 | 0 | 0 | 0 |
| Sham | 0 | 0 | 0 | 0 | 0 | 0 |
| Sham | 0 | 0 | 0 | 0 | 0 | 0 |

**Supplementary 5**

**Table S3.** The original data for short-term neurological severity score (NSS) at different time points among three groups

**Table S4.** The original data for Rotarod Test (S) at different time points among three groups

|  | **Baseline** | **Post-surgery day 1** | **Post-surgery day 2** | **Post-surgery day 3** | **Post-surgery day 4** | **Post-surgery day 7** |
| --- | --- | --- | --- | --- | --- | --- |
| **Semi-ligation** | 189.33 | 10.7 | 32.9 | 64.47 | 70.57 | 34.13 |
| **Semi-ligation** | 166.87 | 92.27 | 4.2 | 92.63 | 98 | 151.97 |
| **Semi-ligation** | 154.33 | 49.67 | 38.6 | 6.9 | 10.43 | 70.83 |
| **Semi-ligation** | 230.5 | 17.83 | 38.87 | 62.4 | 110.9 | 97.23 |
| **Semi-ligation** | 339.5 | 68.1 | 109.8 | 88.77 | 61.1 | 55.53 |
| **Non-semi-ligation** | 255.77 | 134.27 | 129.9 | 104.2 | 152.2 | 135.07 |
| **Non-semi-ligation** | 213.27 | 192.07 | 196.23 | 203.77 | 227.67 | 268.63 |
| **Non-semi-ligation** | 267.4 | 186.33 | 97.63 | 197.07 | 177.6 | 179.97 |
| **Non-semi-ligation** | 160.3 | 116.77 | 154.1 | 206.2 | 188.73 | 208.47 |
| **Non-semi-ligation** | 200.53 | 192.07 | 85.07 | 96.23 | 129.33 | 110.43 |
| **Sham** | 201.03 | 175.5 | 178.93 | 230.1 | 196.6 | 181.5 |
| **Sham** | 168.83 | 229.33 | 181.2 | 134.77 | 197 | 209.07 |
| **Sham** | 200.1 | 263.3 | 225.47 | 232.4 | 256.1 | 179.63 |
| **Sham** | 156.27 | 168.2 | 207.67 | 224.57 | 233.13 | 214.33 |
| **Sham** | 373.4 | 304.7 | 289.37 | 259.43 | 300.37 | 300.43 |

**Table S5.** The original data for long-term neurological severity score (NSS) at different time points among three groups

|  | **Before surgery** | **Post-surgery day 1** | **Post-surgery day 3** | **Post-surgery day 5** | **Post-surgery day 7** | **Post-surgery day 14** |
| --- | --- | --- | --- | --- | --- | --- |
| **Semi-ligation** | 0 | 1 | 3 | 3 | 3 | 2 |
| **Semi-ligation** | 0 | 1 | 1 | 1 | 1 | 1 |
| **Semi-ligation** | 0 | 3 | 3 | 3 | 2 | 2 |
| **Semi-ligation** | 0 | 2 | 2 | 1 | 1 | 0 |
| **Non-semi-ligation** | 0 | 1 | 1 | 1 | 0 | 0 |
| **Non-semi-ligation** | 0 | 2 | 1 | 0 | 0 | 0 |
| **Non-semi-ligation** | 0 | 0 | 0 | 0 | 0 | 0 |
| **Non-semi-ligation** | 0 | 0 | 0 | 0 | 0 | 0 |
| **Sham** | 0 | 0 | 0 | 0 | 0 | 0 |
| **Sham** | 0 | 0 | 0 | 0 | 0 | 0 |
| **Sham** | 0 | 0 | 0 | 0 | 0 | 0 |
| **Sham** | 0 | 0 | 0 | 0 | 0 | 0 |

**Table S6.** The original data for Discrimination Index at different time points among three groups

|  | **Before surgery** | **Post-surgery day 21** | **Post-surgery day 28** |
| --- | --- | --- | --- |
| **Semi-ligation** | 0.63 | 0.57 | 0.39 |
| **Semi-ligation** | 0.67 | 0.54 | 0.56 |
| **Semi-ligation** | 0.63 | 0.39 | 0.56 |
| **Non-semi-ligation** | 0.65 | 0.48 | 0.62 |
| **Non-semi-ligation** | 0.74 | 0.68 | 0.67 |
| **Non-semi-ligation** | 0.55 | 0.48 | 0.36 |
| **Sham** | 0.57 | 0.51 | 0.47 |
| **Sham** | 0.53 | 0.56 | 0.6 |
| **Sham** | 0.52 | 0.55 | 0.6 |

**Table S7.** The original data for Body Weights (g) at different time points among three groups

|  | **Before surgery** | **Post-surgery day 1** | **Post-surgery day 3** | **Post-surgery day 5** | **Post-surgery day 7** | **Post-surgery day 14** | **Post-surgery day 21** | **Post-surgery day 28** |
| --- | --- | --- | --- | --- | --- | --- | --- | --- |
| **Semi-ligation** | 365.8 | 346.7 | 328.2 | 326.4 | 337.7 | 377.7 | 416.6 | 443.8 |
| **Semi-ligation** | 381.9 | 363.8 | 376.5 | 378.1 | 386.4 | 428.7 | 476.2 | 518.7 |
| **Semi-ligation** | 320 | 312.3 | 297 | 290.5 | 284.5 | 297.6 | 343.6 | 396.4 |
| **Semi-ligation** | 296.4 | 277.8 | 282.3 | 282.9 | 282.5 | 327 | 390.7 | 396.2 |
| **Non-semi-ligation** | 289.2 | 260.5 | 256.3 | 254.3 | 248.1 | 277.3 | 279.5 | 286.5 |
| **Non-semi-ligation** | 286.3 | 283.4 | 289.4 | 302.4 | 314.9 | 360.7 | 392.5 | 398.5 |
| **Non-semi-ligation** | 367.6 | 349.5 | 355.9 | 356.6 | 374.8 | 413.5 | 441.4 | 456.3 |
| **Non-semi-ligation** | 316 | 306.7 | 271.8 | 311.1 | 329.9 | 383.5 | 442.9 | 468.2 |
| **Sham** | 340.4 | 337.9 | 313.3 | 325.7 | 338.9 | 406.9 | 450.9 | 490.6 |
| **Sham** | 350.9 | 351.7 | 339 | 364.7 | 373.7 | 437.9 | 480.7 | 537.2 |
| **Sham** | 286.5 | 281.7 | 292 | 295 | 302.8 | 314.8 | 347.8 | 358 |
| **Sham** | 298.3 | 297.1 | 290.8 | 301 | 318.3 | 339.5 | 372.6 | 368.2 |

**Table S8.** The original data for Cumulative mortality rates within 28 days after surgery between semi-ligation and non-semi-ligation groups.

| **Groups** | **Living status** | **Survival time (Day)** |
| --- | --- | --- |
| **Semi-ligation** | Death | 1 |
| **Semi-ligation** | Death | 2 |
| **Semi-ligation** | Alive | 28 |
| **Semi-ligation** | Death | 8 |
| **Semi-ligation** | Alive | 28 |
| **Semi-ligation** | Death | 1 |
| **Semi-ligation** | Death | 1 |
| **Semi-ligation** | Death | 1 |
| **Semi-ligation** | Death | 4 |
| **Semi-ligation** | Death | 3 |
| **Semi-ligation** | Alive | 28 |
| **Semi-ligation** | Death | 1 |
| **Semi-ligation** | Alive | 28 |
| **Semi-ligation** | Death | 14 |
| **Non-semi-ligation** | Alive | 28 |
| **Non-semi-ligation** | Death | 8 |
| **Non-semi-ligation** | Death | 5 |
| **Non-semi-ligation** | Death | 2 |
| **Non-semi-ligation** | Alive | 28 |
| **Non-semi-ligation** | Death | 2 |
| **Non-semi-ligation** | Death | 1 |
| **Non-semi-ligation** | Alive | 28 |
| **Non-semi-ligation** | Alive | 28 |
| **Non-semi-ligation** | Alive | 28 |
| **Non-semi-ligation** | Alive | 28 |
| **Non-semi-ligation** | Death | 2 |
| **Non-semi-ligation** | Alive | 28 |
| **Non-semi-ligation** | Alive | 28 |

**Table S9.** The original data for Thrombus Weights (mg) in one week after surgery among three groups

|  | **Post-surgery day 1** | **Post-surgery day 2** | **Post-surgery day 7** |
| --- | --- | --- | --- |
| **Semi-ligation** | 3.05 | 2.38 | 0.19 |
| **Semi-ligation** | 3.77 | 4.28 | 0.24 |
| **Semi-ligation** | 2.56 | 3.46 | 0.55 |
| **Semi-ligation** | 3.82 | 1.55 | 0.26 |
| **Semi-ligation** | 3.45 | 3.29 | 0 |
| **Non-semi-ligation** | 2.85 | 0.12 | 0 |
| **Non-semi-ligation** | 2.19 | 1.79 | 0 |
| **Non-semi-ligation** | 1.41 | 0.31 | 0 |
| **Non-semi-ligation** | 2.25 | 0.94 | 0 |
| **Non-semi-ligation** | 2.8 | 0.74 | 0 |
| **Sham** | 0 | 0 | 0 |
| **Sham** | 0 | 0 | 0 |
| **Sham** | 0 | 0 | 0 |
| **Sham** | 0 | 0 | 0 |
| **Sham** | 0 | 0 | 0 |

**Table S10.** The original data for Infarction Volumes (mm^3^) in one week after surgery among three groups

|  | **Post-surgery day 1** | **Post-surgery day 2** | **Post-surgery day 7** |
| --- | --- | --- | --- |
| **Semi-ligation** | 94.02 | 513.12 | 145 |
| **Semi-ligation** | 53.75 | 208.88 | 39.7 |
| **Semi-ligation** | 56.9 | 147.69 | 206.08 |
| **Semi-ligation** | 76.45 | 880.04 | 190.44 |
| **Semi-ligation** | 44.28 | 361.93 | 7.73 |
| **Non-semi-ligation** | 0 | 92.21 | 0 |
| **Non-semi-ligation** | 0 | 151.62 | 0 |
| **Non-semi-ligation** | 20.2 | 84.55 | 0 |
| **Non-semi-ligation** | 0 | 0 | 0 |
| **Non-semi-ligation** | 0 | 0 | 0 |
| **Sham** | 0 | 0 | 0 |
| **Sham** | 0 | 0 | 0 |
| **Sham** | 0 | 0 | 0 |
| **Sham** | 0 | 0 | 0 |
| **Sham** | 0 | 0 | 0 |

**Supplementary 6**

**Table S11.** Long-term Neurological severity scores (NSS) at different time points in the semi-ligation, non-semi-ligation and sham groups.

|  | Semi-ligation  (n=4) | Non-semi-ligation  (n=4) | Sham  (n=4) | F | P |
| --- | --- | --- | --- | --- | --- |
| Day 1 | 1.75±0.96 | 0.75±0.96 | 0±0 | 5.045 | 0.034 |
| Day 3 | 2.25±0.96 | 0.50±0.58 | 0±0 | 13.400 | 0.002 |
| Day 5 | 2.00±1.15 | 0.25±0.50 | 0±0 | 9.000 | 0.007 |
| Day 7 | 1.75±0.96 | 0±0 | 0±0 | 13.364 | 0.002 |
| Day 14 | 1.25±0.96 | 0±0 | 0±0 | 6.818 | 0.016 |

Values are expressed as mean ± standard deviation (SD)

The NSS in the semi-ligation group were higher than that in non-semi-ligation and sham groups within one week after surgery. When the assessment was extended to day 14, the NSS in the semi-ligation group was still higher (*P* < 0.05) than that in the other groups.

**Table S12.** The body weights (g) at different time points in the semi-ligation, non-semi-ligation and sham groups.

|  | Semi-ligation  (n=4) | Non-semi-ligation  (n=4) | Sham  (n=4) | F | P |
| --- | --- | --- | --- | --- | --- |
| Before surgery | 341.03±39.66 | 314.78±37.67 | 319.03±31.41 | 0.599 | 0.570 |
| Day 1 | 325.15±38.15 | 300.03±38.00 | 317.10±33.08 | 0.495 | 0.625 |
| Day 3 | 321.00±41.66 | 293.35±43.84 | 308.78±22.65 | 0.552 | 0.594 |
| Day 5 | 319.48±43.44 | 306.10±41.92 | 321.60±31.66 | 0.182 | 0.836 |
| Day 7 | 322.78±49.52 | 316.93±52.47 | 333.43±30.65 | 0.137 | 0.874 |
| Day 14 | 357.75±57.72 | 358.75±58.45 | 374.78±57.32 | 0.109 | 0.898 |
| Day 21 | 406.78±55.27 | 389.08±76.71 | 413.00±62.99 | 0.143 | 0.868 |
| Day 28 | 438.78±57.80 | 402.38±83.03 | 438.50±89.22 | 0.289 | 0.756 |

Values are expressed as mean ± standard deviation (SD)

The body weights of the three groups showed a decreasing trend in one week after surgery. The body weights among the three groups had no significant differences within 28 days after surgery (*P >* 0.05).

**Supplementary 7**

|  | Semi-ligation  (n=5) | Non-semi-ligation  (n=5) | Sham  (n=5) | F | P |
| --- | --- | --- | --- | --- | --- |
| Day 1 | 3.33±0.53 | 2.30±0.58 | 0±0 | 70.361 | 0.000 |
| Day 2 | 2.99±1.05 | 0.78±0.65 | 0±0 | 23.599 | 0.000 |
| Day 7 | 0.25±0.20 | 0±0 | 0±0 | 7.871 | 0.007 |

**Table S13.** Thrombus weight (mg) at different time points in the semi-ligation, non-semi-ligation and sham groups.

Values are expressed as mean ± standard deviation (SD)

On day 1, 2, and 7 after surgery, thrombus weights in the semi-ligation group were significantly heavier than that in the other two groups. There was no thrombosis in the sham group at the corresponding time points.

**Table S14.** Infarction volumes (mm^3^) at different time points in the semi-ligation, non-semi-ligation and sham groups.

|  | Semi-ligation  (n=5) | Non-semi-ligation  (n=5) | Sham  (n=5) | F | P |
| --- | --- | --- | --- | --- | --- |
| Day 1 | 95.08±19.97 | 4.04±9.03 | 0±0 | 41.525 | 0.000 |
| Day 2 | 422.33±292.57 | 65.68±65.33 | 0±0 | 8.621 | 0.005 |
| Day 7 | 117.79±89.48 | 0±0 | 0±0 | 8.665 | 0.005 |

Values are expressed as mean ± standard deviation (SD)

The venous infarction volumes in the semi-ligation group were significantly larger than that in the other two groups on day 1, 2, and 7 after surgery. There was no venous cerebral infarction in the sham group at the corresponding time points.
